# High SURF4 expression is associated with poor prognosis of breast cancer

**DOI:** 10.18632/aging.204409

**Published:** 2022-11-29

**Authors:** Jingtong Zhai, Jiashu Han, Cong Li, Fengzhu Guo, Fei Ma, Binghe Xu

**Affiliations:** 1 Department of Medical Oncology, National Cancer Center/National Clinical Research Center for Cancer/Cancer Hospital, Chinese Academy of Medical Sciences and Peking Union Medical College, Beijing 100021, China; 2 4+4 Medical Doctor Program, Chinese Academy of Medical Sciences and Peking Union Medical College, Beijing 100730, China

**Keywords:** SURF4, breast cancer, prognostic biomarker, overall survival, relapse-free survival

## Abstract

SURF4 has been suggested as an oncogene in cancer. However, the role of SURF4 in breast cancer has not been demonstrated yet. The data were obtained from TCGA database and 1104 patients were analyzed using bioinformatics analysis. SURF4 is significantly (P < 0.001) highly expressed in tumor. High expression of SURF4 was observed in T4, infiltrating ductal carcinoma, ER negative, PR negative, and HER2 positive, female, patients without lymph node metastasis, HER2 overexpression type, and deceased patients. As for characteristics correlated with high expression of SURF4, gender, histological type, molecular subtype, ER, PR, HER2, and vital status exhibited significant differences. The age (HR: 2.317, P < 0.001), stage (HR: 2.090, P < 0.001), and SURF4 expression (HR: 1.958, P = 0.005) exhibited independent prognostic value for overall survival (OS). Patients with high SURF4 expression, higher age, equivocal HER2, higher stages, or positive margin status had shorter OS. The stage (HR: 1.579, P < 0.001), and margin status (HR: 1.463, P = 0.006) exhibited independent prognostic value for relapse-free survival of breast cancer. High expression of SURF4 was first found in breast cancer. High SURF4 expression was observed in breast cancer tissue and cell. SURF4 promoted the proliferation and migration of 4T1 cells. SURF4 may be a biomarker in diagnosis and prognosis of breast cancer.

## INTRODUCTION

Breast cancer is the top malignant tumor that threatens the health of women worldwide with incidence rate of 47.8% and mortality rate of 13.6% [1]. In the past two decades, people have gradually realized that breast cancer is heterogeneous, varying in pathology, genetics, and molecular biology [2, 3]. Breast cancer consists of luminal A, luminal B, human epidermal growth factor receptor 2 (HER2)-enriched, and basal-like molecular subtypes relevant to the clinical practice [4]. Under the guidance of molecular typing, the diagnosis and treatment of breast cancer has gradually entered the era of individualized diagnosis and treatment [5]. The 5-year survival rate and the quality of life of breast cancer patients have been improved [6]. However, some patients will progress to an advanced stage, and advanced breast cancer is still an incurable disease with a median survival of about 24 to 30 months [7, 8].

Early diagnosis has become the key to save the lives of breast cancer patients. Currently, tissue- and serum-based biomarkers are widely used for early-stage tumor screening and to predict disease progression or recurrence [9]. Commonly used biomarkers for breast cancer include estrogen receptor (ER), progesterone receptor (PR), and HER2 [10]. Since the occurrence and progression are heterogeneous, researchers strive to identify novel biomarkers for optimal breast cancer control [11].

Bioinformatic analysis has contributed much to the finding of novel biomarkers, especially in cancer [12]. Kim et al. have suggested SURF4 as an oncogene in cancer [13]. SURF4 is the human homologous gene of the yeast cargo receptor Erv29p with a molecular size of 30 kD [14, 15]. SURF4 contains 7 transmembrane domains and a double-lysine endoplasmic reticulum carboxyl-terminal sequence, which can interact with the ER Golgi Intermediate compartment-53 and p24 protein [16]. However, the role of SURF4 in breast cancer has not been demonstrated yet.

In this study, high expression of SURF4 was first found in breast cancer. Their relationship was further evaluated using bioinformatics analysis. The diagnostic value and independent predictive value in overall survival (OS) and relapse-free survival (RFS) were evaluated. Besides, the nomogram and Gene set enrichment analysis (GSEA) were conducted. The cell and tissue experiments were carried out for final validation.

## RESULTS

### Patient characteristics

In total, 1104 patients were analyzed, 1090 of which (98.73%) were females. As shown in Supplementary Table 1, 589 (53.45%) patients were less than 60 years old, and 790 (71.56%) were diagnosed of infiltrating ductal carcinoma. As for molecular subtype, 142 (12.86%) patients were basal-like type, 67 (6.07%) patients were HER2 overexpression type, 422 (38.22%) patients were luminal A type, 194 (17.57%) patients were luminal B type, and 24 (2.17%) patients were normal-like type. Most patients (56.7%) were in stage II.

### High expression of SURF4 in tumor

**Figure 1:**
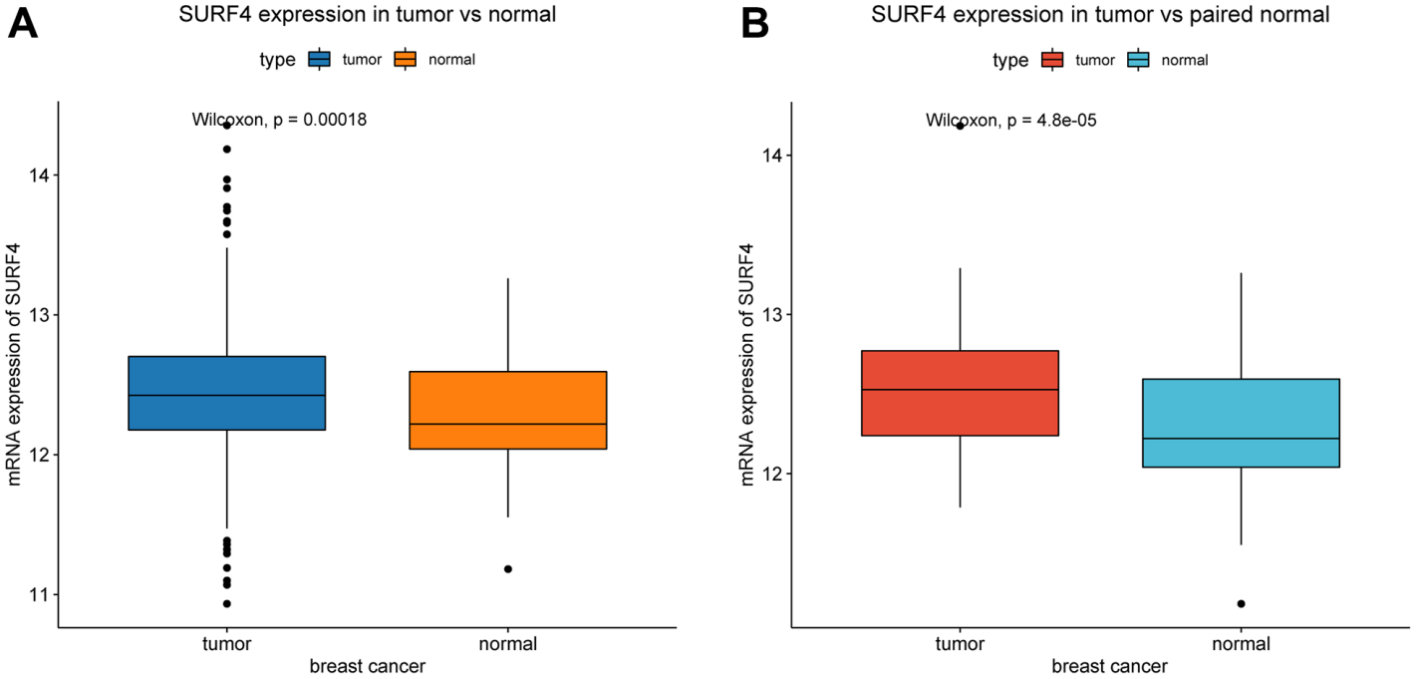
**Expression of SURF4 in breast cancer.** (**A**) The expression of SURF4 in tumor and normal tissues. (**B**) The expression of SURF4 in tumor and paired normal tissues.

Compared with normal tissue, SURF4 is significantly (P < 0.001) highly expressed in tumor (Figure 1A). Besides, SURF4 is significantly (P < 0.001) highly expressed in breast cancer in comparison with paired normal breast tissue (Figure 1B).

**Figure 2:**
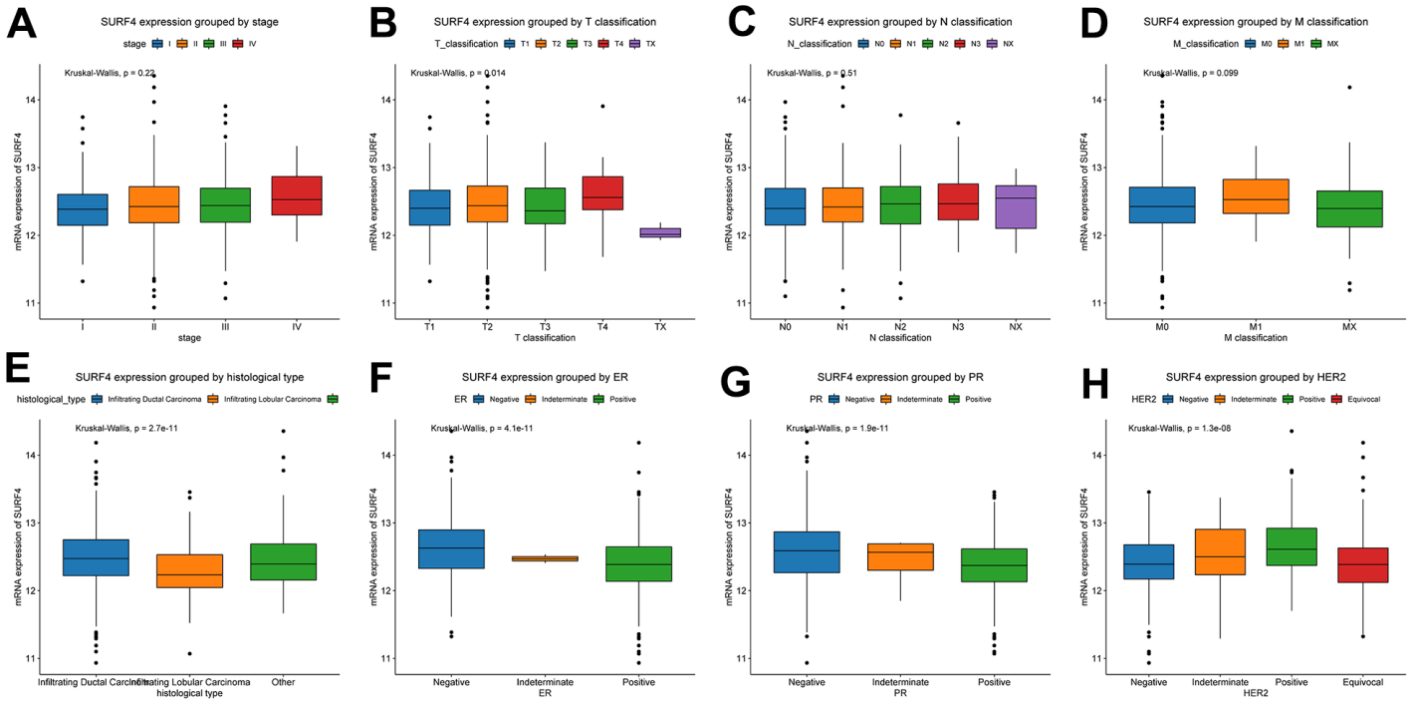
**Expression of SURF4 in breast cancer and its relationship with clinicopathological parameters.** Expression of SURF4 grouped by (**A**) stage, (**B**) T classification, (**C**) N classification, (**D**) M classification, (**E**) histological type, (**F**) ER, (**G**) PR, and (**H**) HER2.

**Figure 3:**
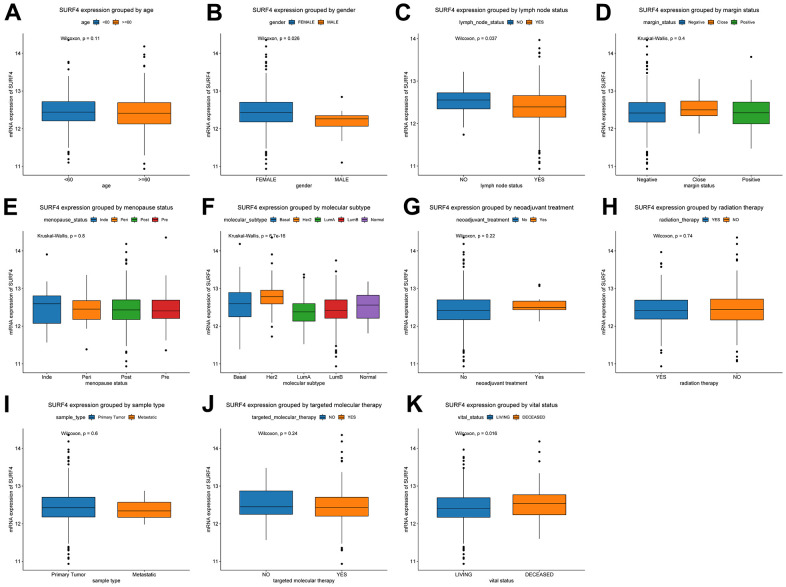
**Expression of SURF4 in breast cancer and its relationship with clinicopathological parameters.** Expression of SURF4 grouped by (**A**) age, (**B**) gender, (**C**) lymph node status, (**D**) margin status, (**E**) menopause status, (**F**) molecular subtype, (**G**) neoadjuvant treatment, (**H**) radiation therapy, (**I**) sample type, (**J**) targeted molecular therapy, and (**K**) vital status.

Furthermore, the expression of SURF4 grouped by distinctive characteristics were evaluated. As shown in Figure 2A–2H, the expression of SURF4 was highest in T4 (P = 0.014), infiltrating ductal carcinoma (P < 0.001), ER negative (P < 0.001), PR negative (P < 0.001), and HER2 positive (P < 0.001) patients. Besides, as shown in Figure 3A–3K, high expression of SURF4 was observed in female (P = 0.026), patients without lymph node metastasis (P = 0.037), HER2 overexpression type (P < 0.001), and deceased patients (P = 0.016). Other characteristics showed no significantly statistical differences.

### Diagnostic value of SURF4 expression for breast cancer

The receiver operating characteristic (ROC) curves were plotted to evaluate the diagnostic value of SURF4 expression for breast cancer. As shown in Supplementary Figure 1A, the cut-off value between normal and tumor was 12.241, and the AUC was 0.606. Besides, the AUC for stage I-IV (Supplementary Figure 1B–1E) was 0.581, 0.609, 0.613, and 0.693, respectively.

### Characteristics correlated with high expression of SURF4

First, the patients were divided into the low and high SURF4 expression groups according to the cut-off value between normal and tumor obtained from ROC curves. Then, the characteristics correlated with high SURF4 expression were studied. As shown in Supplementary Table 2, gender (P = 0.032), histological type (P < 0.001), molecular subtype (P < 0.001), ER (P < 0.001), PR (P < 0.001), HER2 (P < 0.001), and vital status (P = 0.002) exhibited significant differences. However, other characteristics showed no significant differences.

### High SURF4 expression is correlated with poor OS

**Figure 4:**
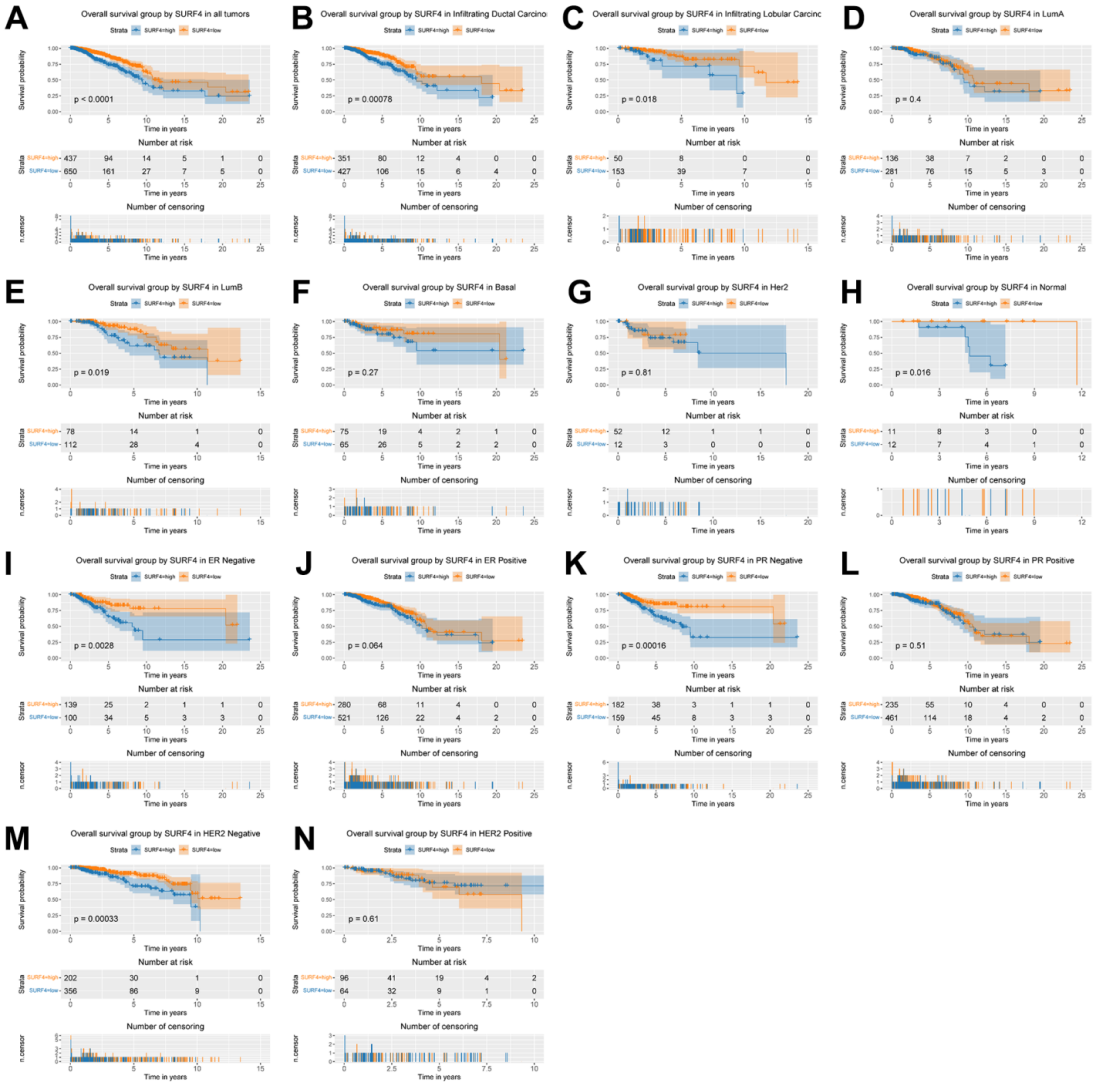
**Kaplan-Meier curve for OS group by SURF4.** (**A**) SURF4 in all tumors. (**B**–**H**) Subgroup analysis according to typing in infiltrating ductal carcinoma, infiltrating lobular carcinoma, Luminal A, Luminal B, basal-like, HER2 overexpression, normal-like. (**I**–**N**) Subgroup analysis according to status in ER negative, ER positive, PR negative, PR positive, HER2 negative, and HER2 positive.

Kaplan–Meier curves were used to evaluate the OS correlated with SURF4 expression. As shown in Figure 4A, high SURF4 expression was significantly correlated with poor OS (P < 0.001). Moreover, high SURF4 expression was correlated with poor OS of breast cancer patients in infiltrating ductal carcinoma (P < 0.001), infiltrating lobular carcinoma (P = 0.018), luminal B type (P = 0.019), normal-like type (P = 0.016), ER negative (P = 0.003), PR negative (P < 0.001), and HER2 negative (P < 0.001) by subgroup analysis (Figure 4B–4N).

**Figure 5:**
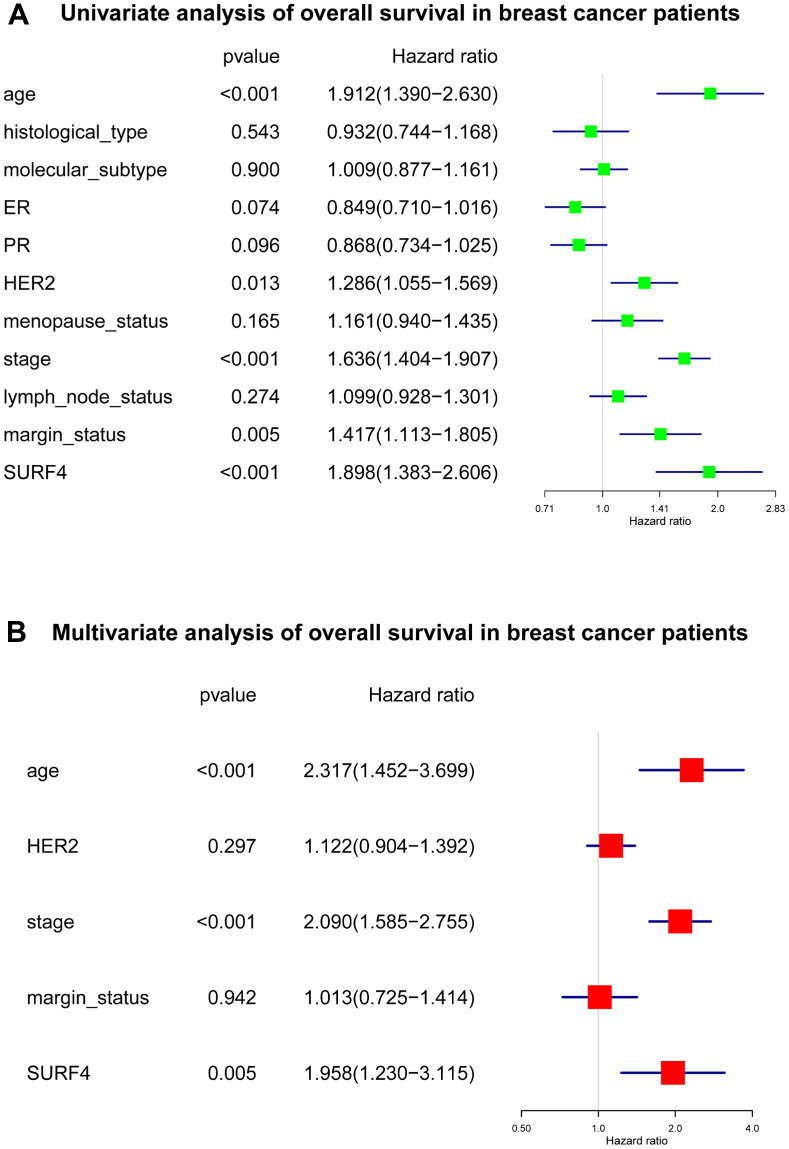
**Forest plot of Cox regression analysis about SURF4 and OS.** (**A**) Univariate analysis of OS in breast cancer patients. (**B**) Multivariate analysis of OS in breast cancer patients.

As shown in Figure 5 and Supplementary Table 3, several important variables were identified by univariate analysis, and confirmed by the subsequent multivariate analysis. The age [hazard ratio (HR): 2.317, 95% confidence interval (CI): 1.452-3.699, P < 0.001], stage (HR: 2.090, 95% CI: 1.585-2.755, P < 0.001), and SURF4 expression (HR: 1.958, 95% CI: 1.230-3.115, P = 0.005) exhibited independent prognostic value for OS of breast cancer.

### High SURF4 expression is correlated with poor RFS

**Figure 6:**
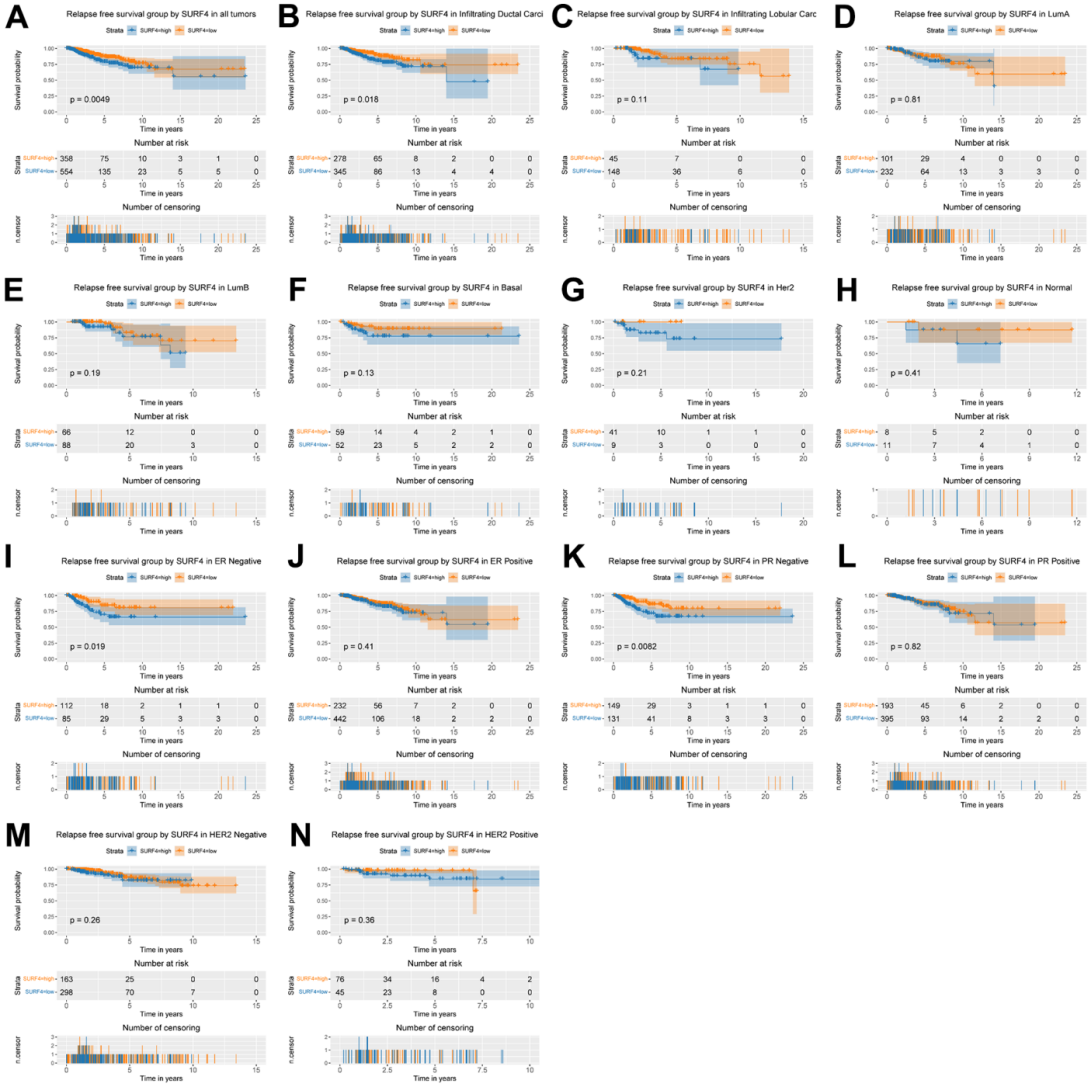
**Kaplan-Meier curve for RFS group by SURF4.** (**A**) SURF4 in all tumors. (**B**–**H**) Subgroup analysis according to typing in infiltrating ductal carcinoma, infiltrating lobular carcinoma, Luminal A, Luminal B, basal-like, HER2 overexpression, normal-like. (**I**–**N**) Subgroup analysis according to status in ER negative, ER positive, PR negative, PR positive, HER2 negative, and HER2 positive.

As shown in Figure 6A, high SURF4 expression was significantly correlated with poor RFS (P = 0.005). Moreover, high SURF4 expression was correlated with poor RFS of breast cancer patients in infiltrating ductal carcinoma (P = 0.018), ER negative (P = 0.019), PR negative (P = 0.008) by subgroup analysis (Figure 6B–6N).

**Figure 7:**
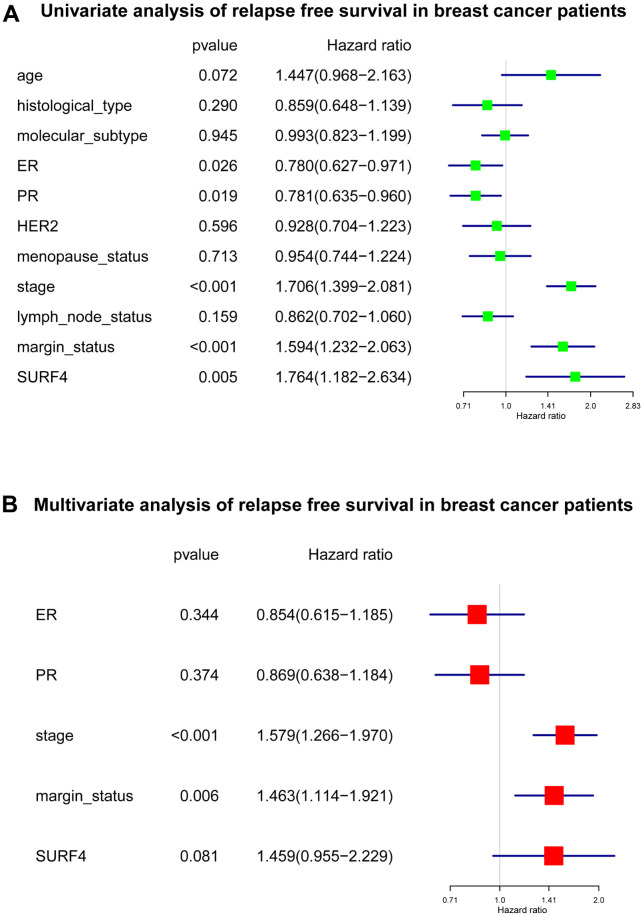
**Forest plot of Cox regression analysis about SURF4 and RFS.** (**A**) Univariate analysis of RFS in breast cancer patients. (**B**) Multivariate analysis of RFS in breast cancer patients.

As shown in Figure 7 and Supplementary Table 4, several important variables were identified by univariate analysis, and confirmed by the subsequent multivariate analysis. The stage (HR: 1.579, 95% CI: 1.266-1.970, P < 0.001), and margin status (HR: 1.463, 95% CI: 1.114-1.921, P = 0.006) exhibited independent prognostic value for RFS of breast cancer.

### Predictive value of SURF4 for survival

**Figure 8:**
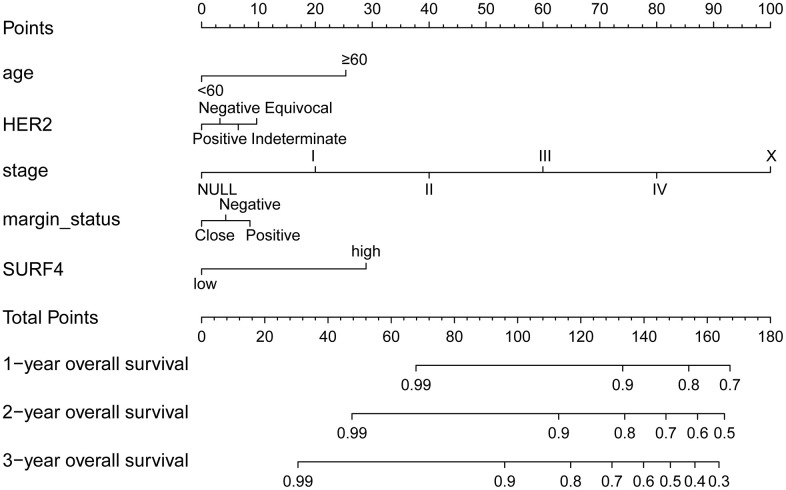
**The nomogram about SURF4 and OS.** 1-, 3- and 5-year related survival probabilities were obtained by drawing a line straight down to the risk axis.

The nomograms of SURF4 were generated to predicate survival of breast cancer patients. Patients with high SURF4 expression showed shorter OS (Figure 8). Besides, the patients with higher age, equivocal HER2, higher stages, or positive margin status also had shorter OS.

### SURF4 related signaling pathways by GSEA

**Figure 9:**
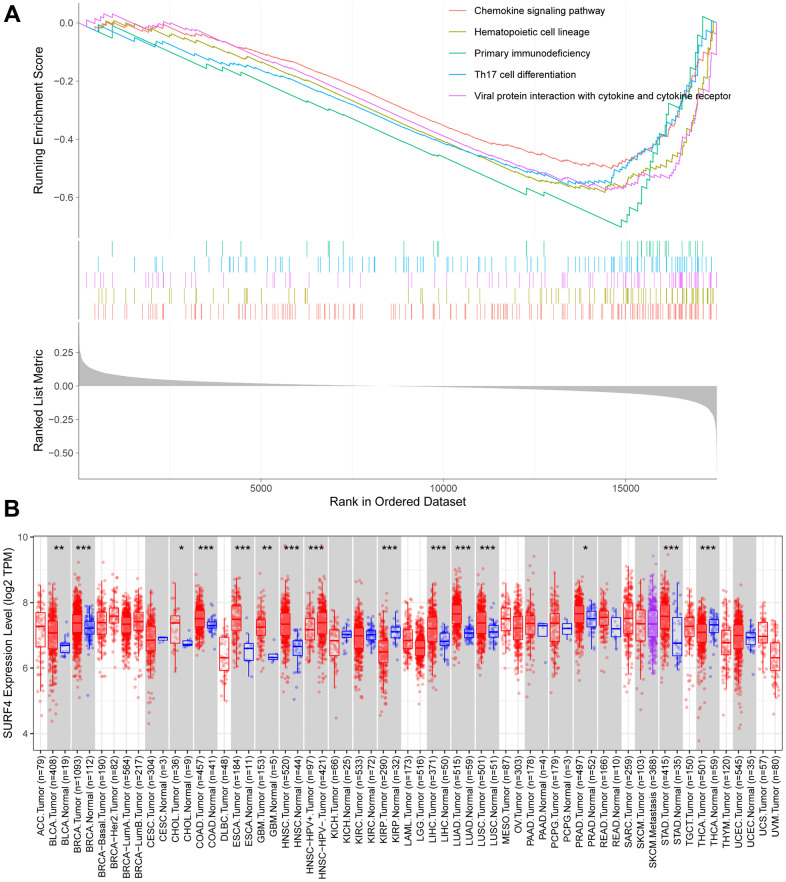
**Expression of SURF4 by enrichment analysis and in TIMER database.** (**A**) Top 5 enrichments with enriched high expression of SURF4. (**B**) Expression of SURF4 in diverse types of human cancers in the TIMER database.

As shown in Supplementary Table 5, the GSEA analysis was further performed to evaluate the SURF4 related signaling pathways. The top 5 signaling pathways were shown in Figure 9A. The results showed that SURF4 may influence the development of breast cancer by controlling chemokine signaling pathway, Th17 cell differentiation, primary immunodeficiency, etc.

### Expression of SURF4 in TIMER database

The expressions of SURF4 in different cancers were further studied using TIMER database (Figure 9B). Significant high SURF4 expression was found in bladder urothelial carcinoma, etc.

### High SURF4 expression in breast cancer tissue and cell

**Figure 10:**
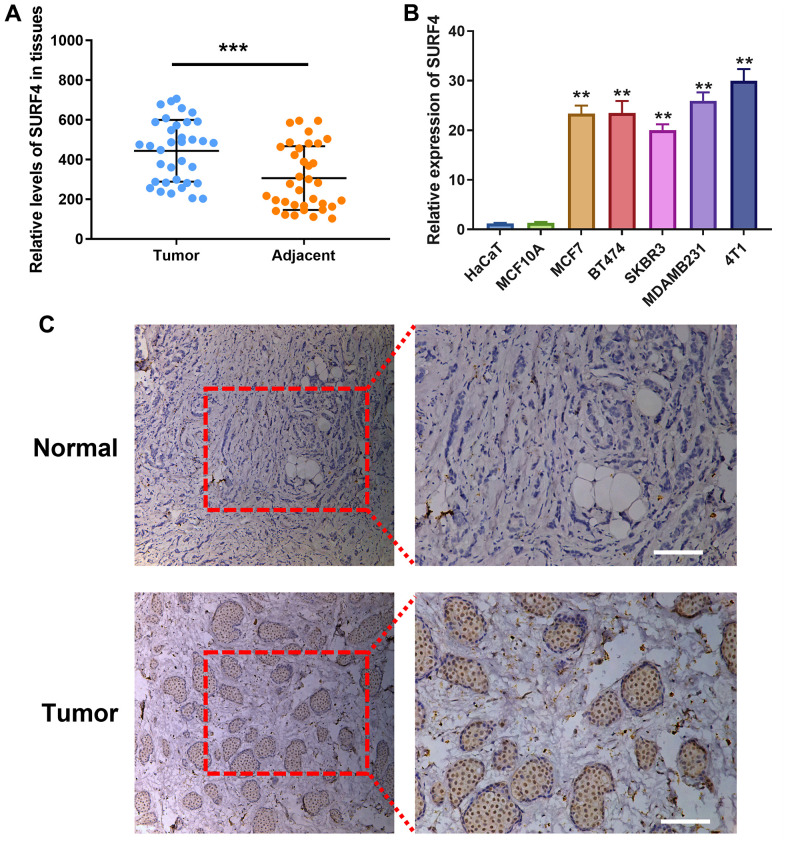
**Expression of SURF4 in human breast tissues and cell lines.** (**A**) SURF4 expression in tumor and adjacent normal tissue by qRT-PCR (N = 35). (**B**) SURF4 expression in HaCaT (Human immortalized epidermal cell), MCF10A (Human normal breast cell), MCF7 (Human luminal A type breast cancer cell), BT474 (Human luminal B type breast cancer cell), SKBR3 (Human HER2 overexpression type breast cancer cell), MDAMB231 and 4T1 (Human triple-negative breast cancer cell) by qRT-PCR in triplicate. (**C**) SURF4 expression in normal and tumor tissue by IHC. The tissues were from single patient. Scale bar = 100 μm. **P < 0.01; ***P < 0.001.

As shown in Figure 10A, SURF4 expression was significantly higher in tumor than adjacent normal tissue (P < 0.001). As shown in Figure 10B, significant high SURF4 expression (P < 0.01) was observed in MCF7 (Human luminal A type breast cancer cell), BT474 (Human luminal B type breast cancer cell), SKBR3 (Human HER2 overexpression type breast cancer cell), MDAMB231 and 4T1 (Human triple-negative breast cancer cell). Meanwhile, HaCaT (Human immortalized epidermal cell) and MCF10A (Human normal breast cell) showed much lower SURF4 expression. Importantly, 4T1 cell line showed the highest SURF4 expression and used subsequently for *in vitro* experiment. The high SURF4 expression in breast cancer tissue was further validated by immunohistochemistry (IHC) (Figure 10C).

### SURF4 promoted the proliferation and migration of 4T1 cells

**Figure 11:**
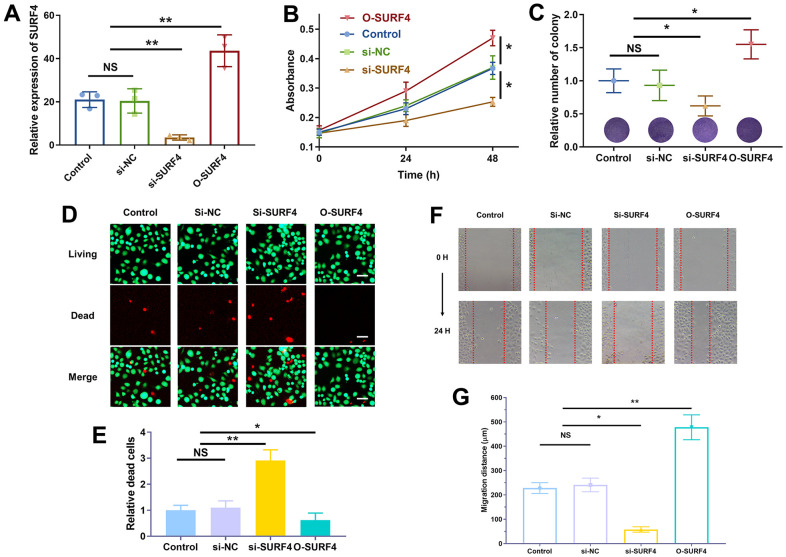
**SURF4 promoted cell proliferation and migration of breast cancer cell 4T1.** (**A**) Efficiency of plasmid transfection by qRT-PCR. (**B**) The cell viability by CCK8 assay. (**C**) Cell proliferation ability by colony formation assay. (**D**, **E**) Cell proliferation ability by living/dead staining assay. Scale bar = 50 μm. (**F**, **G**) Cell migration ability by wound healing assay. The experiments were repeated for 3 times. 40 x under light microscopy. NS, no significance; *P < 0.05; **P < 0.01.

The successful over-expression (O-SURF4) and knockdown (si-SURF4) of SURF4 was shown in Figure 11A. As shown in Figure 11B, si-SURF4 group showed decreased cell proliferation (P < 0.01), while O-SURF4 group showed increased cell proliferation (P < 0.01). The results of colony formation (Figure 11C) and living/dead cell staining (Figure 11D, 11E) were consistent. Si-SURF4 group showed fewer colonies (P < 0.05) and more dead cells (P < 0.01), while O-SURF4 group showed more colonies (P < 0.05) and fewer dead cells (P < 0.05). Finally, the migration distance was shorter in si-SURF4 group (P < 0.05) and longer in O-SURF4 group (Figure 11F, 11G, P < 0.01). The results indicated that SURF4 promoted the proliferation and migration of 4T1 cells.

## DISCUSSION

In 2020, the incidence of breast cancer reached 11.7%, surpassing lung cancer for the first time to become the world’s most commonly diagnosed cancer. Continuous exploration of earlier diagnosis methods has become the most urgent task [17]. Breast cancer is a very heterogeneous disease [18]. It has become widely accepted that breast cancer can be classified according to molecular markers. In 2001, Peru et al. applied complementary DNA microarray technology to detect postoperative specimens, and divided breast cancer into different subtypes with different clinical prognosis, namely luminal A, luminal B, basal-like, HER2 overexpression, and normal-like type [19].

Diverse types of breast cancer have different biological characteristics and treatment methods. Hormone receptor-positive luminal A and luminal B have the highest proportions in breast cancer, and can be treated with endoprostheses with relatively good prognosis [20]. The 5-year selective ER modulator tamoxifen therapy can reduce the recurrence rate by about 50% and the mortality by about 30% [21]. Compared with luminal A type, luminal B type has the following characteristics: lower expression levels of estrogen or estrogen-related genes, low or no expression of PR, higher grade of tumor and higher expression levels of proliferation-related genes, growth factor receptor pathways such as IGF-1R and PI3K/AKT/mTOR are easily activated, etc. [22]. HER2 overexpression type has poor biological behavior and high recurrence rate, but its treatment has made significant progress with the application of targeted drugs such as trastuzumab, pertuzumab, and lapatinib, etc. [5, 23]. The basal-like type lacking hormone receptors and overexpressing HER2 is often considered triple-negative breast cancer, which can only be treated with chemotherapy, and is more common in patients with *BRCA1* mutations or African ancestry [24, 25].

Our results indicated that SURF4 was significantly (P < 0.001) highly expressed in tumor. High expression of SURF4 was observed in T4, infiltrating ductal carcinoma, ER negative, PR negative, HER2 positive, female, patients without lymph node metastasis, HER2 overexpression type, and deceased patients. The diagnostic ability increased with stage increased as the AUC for stage I-IV was 0.581, 0.609, 0.613, and 0.693, respectively. The age, stage, and SURF4 expression exhibited independent prognostic value for OS of breast cancer. Patients with high SURF4 expression, higher age, equivocal HER2, higher stages, or positive margin status had shorter OS. The stage and margin status exhibited independent prognostic value for RFS of breast cancer.

As is known, Ki-67 is a nuclear antigen associated with proliferating cells, and currently a widely used tumor cell proliferation activity marker [26]. Ki-67 expression level is significantly higher in malignant tumors [27]. Ki-67 has been used as one of the proliferation markers in early breast cancer [28]. Relevant studies have shown that changes in the expression level of Ki-67 can be used as a sensitivity indicator to predict the efficacy of neoadjuvant endocrine therapy [29]. Neoadjuvant endocrine therapy trials IMPACT and Z1031 showed a survival benefit after inhibition of Ki-67 expression [30, 31]. Neoadjuvant endocrine therapy has been used as the primary endpoint, but its value in predicting the efficacy of neoadjuvant chemotherapy remains controversial [32].

Limited studies have showed the role of SURF4 in cancer. Yue et al. found SURF4 possessed the ability for maintaining stemness of ovarian cancer, and may serve as a potential target [33]. Kim et al. reported that SURF4 could induce cellular transformation and cell migration *in vitro* and has oncogenic transformation ability *in vivo* [13]. Our study reported a novel biomarker SURF4 in breast cancer. High SURF4 expression was confirmed in breast cancer tissue and cells. SURF4 expression was significantly higher in tumor. The high SURF4 expression in breast cancer tissue was further validated by IHC. Besides, significant high SURF4 expression (P < 0.01) was observed in MCF7 (Human luminal A type breast cancer cell), BT474 (Human luminal B type breast cancer cell), SKBR3 (Human HER2 overexpression type breast cancer cell), MDAMB231 and 4T1 (Human triple-negative breast cancer cell). The *in vitro* experiments suggested SURF4 promoted the proliferation and migration of 4T1 cells. Combing the results of GSEA analysis, SURF4 may influence the development of breast cancer by controlling chemokine signaling pathway, etc. However, the underlying mechanisms need to be studied in the future. Also, the *in vivo* experiments can improve the evidence power of the findings.

In conclusion, high expression of SURF4 was first found in breast cancer. SURF4 expression exhibited independent prognostic value for OS, and patients with high SURF4 expression had shorter OS. SURF4 promoted the proliferation and migration of 4T1 cells. SURF4 may be a biomarker to play a role in diagnosis and prognosis of breast cancer. Our findings may indicate SURF4 as a novel therapeutic target for treatment of breast cancer.

## MATERIALS AND METHODS

### Data processing and comparison

The files of mRNA expression and associated clinical data were acquired from the The Cancer Genome Atlas (TCGA) database [34]. Non-parametric rank sum tests were used to evaluate the SURF4 mRNA expression. Wilcoxon rank sum tests and Kruskal-Wallis tests were used to compare two and multiple subgroups, respectively. Chi-square tests along with Fisher’s exact tests were used to evaluate the characteristics correlated with SURF4 expression.

### Diagnostic value evaluation

The pROC program was used for visualization of ROC curves, which were plotted to evaluate the diagnostic value of SURF4 [35]. According to the cut-off value obtained from ROC curves between normal and tumor, the patients were further divided into the low and high SURF4 expression groups. The area under the ROC curves (AUC) was also calculated.

### Survival evaluation and nomogram plotting

The Kaplan-Meier curves were used to analyze the OS and RFS by the R survival package [36]. To investigate the independent predictive ability of SURF4 in breast cancer, univariate and multivariate Cox analysis were carried out. According to SURF4 expression, the patients with breast cancer were divided into groups. Based on different SURF4 expression, the age, HER2, stage, margin status, and 1,3,5-year OS were compared.

### GSEA analysis and TIMER database mining

To investigate the relation between SURF4 expression and enriched signaling pathways, the TCGA database was first searched, and then GSEA analysis was performed. The TIMER database was used for study of SURF4 expression in different cancers.

### Sample collection

Breast cancer and adjacent tissues were obtained from 35 subjects, and placed in liquid nitrogen immediately after resection. The study was approved by the institutional ethical committee and conformed to the Declaration of Helsinki.

### Cell culture and plasmid transfection

The cells were cultured in 1640 medium containing 10% fetal bovine serum, and transfected with the si-NC (negative control), si-SURF4 (small-interfering RNAs against SURF4) and O-SURF4(overexpressed SURF4) plasmids. All the plasmids were purchased from Genepharma (Shanghai, China).

### Real-time quantitative PCR

Total RNA was extracted using TRIzol (Invitrogen, USA). 1 mL of isolated RNA was used for the reverse transcription. The real-time quantitative PCR (RT-qPCR) procedure was then completed. The experiments were repeated for 3 times. The primers are as follows: SURF4 (Forward: 5′-CCTTTAAGGCTTGGCCTACG-3′; Reverse: 5′- GGGCCAGGTTCCTCATCAAA-3′), and β-actin (Forward: 5′-GGAGCGAGATCCCTCCAAAAT-3′; Reverse: 5′-GGCTGTTGTCATACTTCTCATGG-3′).

### Immunohistochemistry staining

The tissues were from single patient. The immunohistochemistry (IHC) staining was performed according to the manual instructions (#13079, Cell Signaling Technology, MA, USA). Anti-SURF4 primary antibody (ab133369, Abcam company, Shanghai, China) and corresponding secondary antibody (rabbit) were used. The fluorescence microscope was used for imaging.

### Cell proliferation and migration assay

After a 24-hour culture period with different plasmids, the absorbance at 450 nm was measured after the addition of 10 μL of CCK-8 reagent (CK04, Dojindo company, Beijing, China) [37]. After being scraped to create a 1-mm gap, the cells treated with different plasmids were grown for 48 hour, and images were obtained at 0 and 48 hours to record the migration distance [38]. The experiments were repeated for 3 times.

### Statistical analysis

R3.5.1 was used to perform bioinformatics analysis [39]. The Kaplan-Meier curve was used to analyze the survival rate [40]. To investigate the independent predictive capability of SURF4 in breast cancer, univariate and multivariate Cox analyses were used. It was statistically significant at P < 0.05.

## Supplementary Material

Supplementary Figure 1

Supplementary Tables
