# Supplementary material for: High SURF4 expression is associated with poor prognosis of breast cancer
Source: Aging (Albany NY). 2022 Nov 29;14(22):9317–37. doi: 10.18632/aging.204409 (PMC9740377; doi:10.18632/aging.204409)
Supplement: Supplementary Figure 1 [file aging-14-204409-s001.pdf]

SUPPLEMENTARY FIGURE

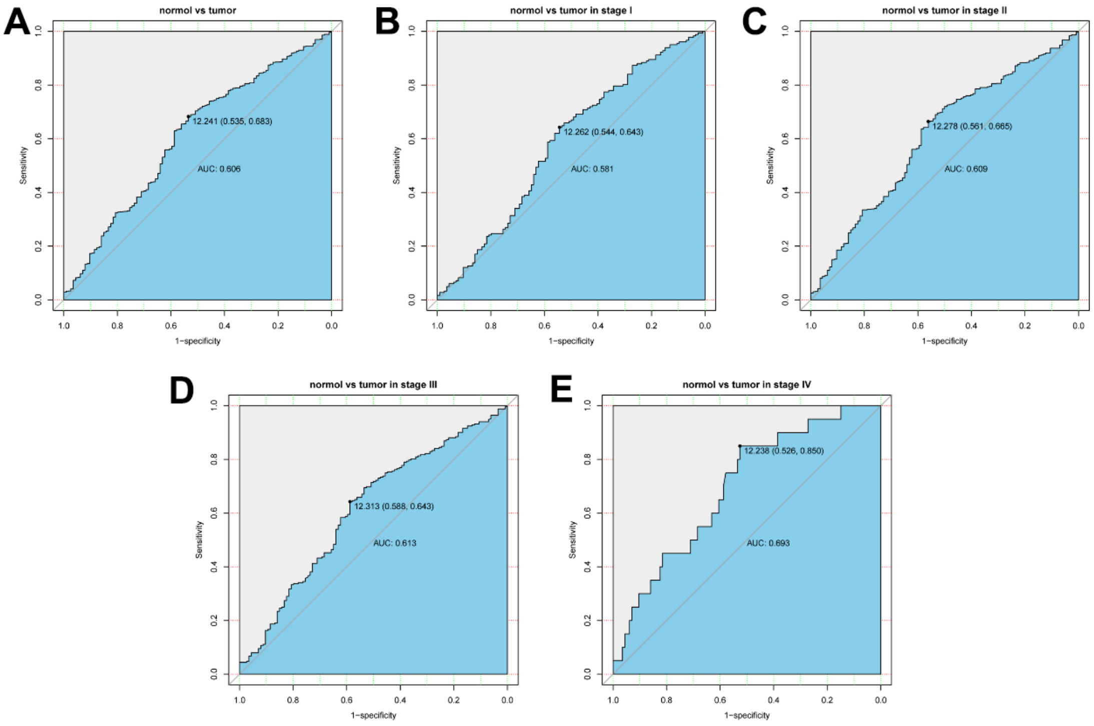

Supplementary Figure 1. ROC curve to assess the diagnostic value of SURF4. (**A**) ROC curve of SURF4 in normal vs. tumor. (**B**–**E**) ROC curve of SURF4 in stage I, stage II, stage III, and stage IV.
