# Supplementary material for: High SURF4 expression is associated with poor prognosis of breast cancer
Source: Aging (Albany NY). 2022 Nov 29;14(22):9317–37. doi: 10.18632/aging.204409 (PMC9740377; doi:10.18632/aging.204409)
Supplement: Supplementary Tables [file aging-14-204409-s002.pdf]

SUPPLEMENTARY TABLES

Supplementary Table 1. Patient characteristics.

Characteristics	Numbers of cases
Age	
<60	589(53.45)
>=60	513(46.55)
Gender	
NA	2(0.18)
Female	1090(98.73)
Male	12(1.09)
Histological type	
NA	3(0.27)
Infiltrating Ductal Carcinoma	790(71.56)
Infiltrating Lobular Carcinoma	204(18.48)
Other	107(9.69)
Molecular subtype	
NA	255(23.10)
Basal	142(12.86)
HER2	67(6.07)
LuminalA	422(38.22)
LuminalB	194(17.57)
Normal	24(2.17)
ER	
NA	50(4.53)
Indeterminate	2(0.18)
Negative	239(21.65)
Positive	813(73.64)
PR	
NA	51(4.62)
Indeterminate	4(0.36)
Negative	345(31.25)
Positive	704(63.77)
HER2	
NA	183(16.58)
Equivocal	180(16.30)
Indeterminate	12(1.09)
Negative	565(51.18)
Positive	164(14.86)
Menopause status	
NA	93(8.42)
Inde	34(3.08)
Peri	40(3.62)
Post	706(63.95)
Pre	231(20.92)
T classification	
NA	2(0.18)
T1	281(25.45)
T2	640(57.97)
T3	138(12.5)
T4	40(3.62)
TX	3(0.27)
N classification	

NA	2(0.18)
N0	516(46.74)
N1	367(33.24)
N2	120(10.87)
N3	79(7.16)
NX	20(1.81)
M classification	
NA	2(0.18)
M0	917(83.06)
M1	22(1.99)
MX	163(14.76)
Stage	
NA	10(0.91)
I	182(16.49)
II	626(56.70)
III	252(22.83)
IV	20(1.81)
X	14(1.27)
Lymph node status	
NA	379(34.33)
No	28(2.54)
Yes	697(63.13)
Margin status	
NA	72(6.52)
Close	31(2.81)
Negative	922(83.51)
Positive	79(7.16)
Vital status	
NA	2(0.18)
Deceased	155(14.04)
Living	947(85.78)
Radiation therapy	
NA	102(9.24)
No	445(40.31)
Yes	557(50.45)
Neoadjuvant treatment	
NA	3(0.27)
No	1088(98.55)
Yes	13(1.18)
Targeted molecular therapy	
NA	525(47.55)
No	46(4.17)
Yes	533(48.28)
Sample type	
Metastatic	7(0.63)
Primary Tumor	1097(99.37)
SURF4	
high	445(40.31)
low	659(59.69)

Note: ER, estrogen receptor; PR, progesterone receptor; HER2, human epidermal growth factor receptor 2; NA, not available; X represents uncertain.

Supplementary Table 2. Characteristics correlated with SURF4 expression.

Parameter	Variable	N	SURF4 mRNA expression				χ2	P value
			high	%	low	%		
Age	<60	589	247	(55.76)	342	(51.90)	1.586	0.210
	>=60	513	196	(44.24)	317	(48.10)		
Gender	Female	1090	442	(99.77)	648	(98.33)	5.125	**0.032**
	Male	12	1	(0.23)	11	(1.67)		
Histological type	Infiltrating Ductal Carcinoma	790	355	(80.32)	435	(66.01)	28.903	**<0.001**
	Infiltrating Lobular Carcinoma	204	51	(11.54)	153	(23.22)		
	Other	107	36	(8.14)	71	(10.77)		
Molecular subtype	Basal	142	76	(21.23)	66	(13.44)	65.143	**<0.001**
	HER22	67	54	(15.08)	13	(2.65)		
	LuminalA	422	137	(38.27)	285	(58.04)		
	LumialB	194	79	(22.07)	115	(23.42)		
	Normal	24	12	(3.35)	12	(2.44)		
ER	Indeterminate	2	1	(0.24)	1	(0.16)	41.049	**<0.001**
	Negative	239	139	(32.71)	100	(15.90)		
	Positive	813	285	(67.06)	528	(83.94)		
PR	Indeterminate	4	2	(0.47)	2	(0.32)	38.503	**<0.001**
	Negative	345	185	(43.63)	160	(25.44)		
	Positive	704	237	(55.90)	467	(74.24)		
HER2	Equivocal	180	63	(16.94)	117	(21.31)	32.137	**<0.001**
	Indeterminate	12	6	(1.61)	6	(1.09)		
	Negative	565	205	(55.11)	360	(65.57)		
	Positive	164	98	(26.34)	66	(12.02)		
Menopause status	Inde	34	19	(4.65)	15	(2.49)	3.592	0.311
	Peri	40	17	(4.16)	23	(3.82)		
	Post	706	281	(68.70)	425	(70.60)		
	Pre	231	92	(22.49)	139	(23.09)		
T classification	T1	281	103	(23.25)	178	(27.01)	7.354	0.133
	T2	640	268	(60.50)	372	(56.45)		
	T3	138	51	(11.51)	87	(13.20)		
	T4	40	21	(4.74)	19	(2.88)		
	TX	3	0	(0.00)	3	(0.46)		
N classification	N0	516	199	(44.92)	317	(48.10)	3.294	0.513
	N1	367	145	(32.73)	222	(33.69)		
	N2	120	55	(12.42)	65	(9.86)		
	N3	79	34	(7.67)	45	(6.83)		
	NX	20	10	(2.26)	10	(1.52)		
M classification	M0	917	372	(83.97)	545	(82.70)	1.710	0.414
	M1	22	11	(2.48)	11	(1.67)		
	MX	163	60	(13.54)	103	(15.63)		
Stage	I	182	62	(14.12)	120	(18.32)	4.441	0.335
	II	626	253	(57.63)	373	(56.95)		
	III	252	108	(24.60)	144	(21.98)		
	IV	20	10	(2.28)	10	(1.53)		
	X	14	6	(1.37)	8	(1.22)		
Lymph node status	No	28	15	(5.47)	13	(2.88)	3.084	0.106
	Yes	697	259	(94.53)	438	(97.12)		
Margin status	Close	31	14	(3.42)	17	(2.73)	0.680	0.692
	Negative	922	366	(89.49)	556	(89.25)		
	Positive	79	29	(7.09)	50	(8.03)		
Vital status	Deceased	155	81	(18.28)	74	(11.23)	10.910	**0.002**
	Living	947	362	(81.72)	585	(88.77)		

Radiation therapy	No	445	190	(47.03)	255	(42.64)	1.880	0.186
	Yes	557	214	(52.97)	343	(57.36)		
Neoadjuvant treatment	No	1088	437	(98.65)	651	(98.94)	0.192	0.785
	Yes	13	6	(1.35)	7	(1.06)		
Targeted molecular therapy	No	46	22	(9.09)	24	(7.12)	0.747	0.454
	Yes	533	220	(90.91)	313	(92.88)		
Sample type	Metastatic	7	2	(0.45)	5	(0.76)	0.403	0.707
	Primary Tumor	1097	443	(99.55)	654	(99.24)		

Note: ER, estrogen receptor; PR, progesterone receptor; HER2, human epidermal growth factor receptor 2; NA, not available; X represents uncertain.

Supplementary Table 3. Univariate and multivariate analysis for overall survival.

Parameters	Univariate analysis			Multivariate analysis		
	HR	95% CI	P value	HR	95% CI	P value
Age	1.91	1.39-2.63	<0.001	2.32	1.45-3.70	**<0.001**
ER	0.85	0.71-1.02	0.074			
HER2	1.29	1.05-1.57	0.013	1.12	0.90-1.39	0.297
Histological type	0.93	0.74-1.17	0.543			
Lymph node status	1.10	0.93-1.30	0.274			
Margin status	1.42	1.11-1.81	0.005	1.01	0.72-1.41	0.942
Menopause status	1.16	0.94-1.43	0.165			
Molecular subtype	1.01	0.88-1.16	0.901			
PR	0.87	0.73-1.03	0.096			
Stage	1.64	1.40-1.91	<0.001	2.09	1.59-2.75	**<0.001**
SURF4	1.90	1.38-2.61	<0.001	1.96	1.23-3.11	**0.005**

Note: ER, estrogen receptor; PR, progesterone receptor; HER2, human epidermal growth factor receptor 2; HR, hazard ratio; CI, confidence interval.

Supplementary Table 4. Univariate and multivariate analysis for relapse-free survival.

Parameters	Univariate analysis			Multivariate analysis		
	HR	95% CI	P value	HR	95% CI	P value
Age	1.45	0.97-2.16	0.072			
ER	0.78	0.63-0.97	0.026	0.85	0.61-1.18	0.344
HER2	0.93	0.70-1.22	0.596			
Histological type	0.86	0.65-1.14	0.290			
Lymph node status	0.86	0.70-1.06	0.159			
Margin status	1.59	1.23-2.06	<0.001	1.46	1.11-1.92	**0.006**
Menopause status	0.95	0.74-1.22	0.713			
Molecular subtype	0.99	0.82-1.20	0.945			
PR	0.78	0.64-0.96	0.019	0.87	0.64-1.18	0.374
Stage	1.71	1.40-2.08	<0.001	1.58	1.27-1.97	**<0.001**
SURF4	1.76	1.18-2.63	0.005	1.46	0.95-2.23	0.081

Note: ER, estrogen receptor; PR, progesterone receptor; HER2, human epidermal growth factor receptor 2; HR, hazard ratio; CI, confidence interval.

Supplementary Table 5. Enriched signaling pathways by GSEA analysis.

Description	Set size	Enrichment score	NES	P value	P adjusted value	Q value
Chemokine signaling pathway	188	-0.5014	-1.9249	1.9E-07	6.4E-05	5.3E-05
Hematopoietic cell lineage	94	-0.5834	-2.0432	5.9E-07	9.9E-05	8.1E-05
Viral protein interaction with cytokine and cytokine receptor	95	-0.5749	-2.0224	1.4E-06	0.00016	0.00013
Th17 cell differentiation	106	-0.5569	-1.9797	2.8E-06	0.00024	0.00019
Primary immunodeficiency	36	-0.703	-2.0913	3.9E-06	0.00026	0.00021
Cytokine-cytokine receptor interaction	282	-0.4153	-1.6693	1.1E-05	0.00056	0.00046
Cell cycle	119	0.47578	1.88178	1.2E-05	0.00056	0.00046
Intestinal immune network for IgA production	45	-0.647	-2.003	1.6E-05	0.00058	0.00048
B cell receptor signaling pathway	80	-0.5554	-1.9029	1.6E-05	0.00058	0.00048
Osteoclast differentiation	127	-0.503	-1.8477	1.7E-05	0.00058	0.00048
Systemic lupus erythematosus	50	-0.6302	-1.9953	2.4E-05	0.00072	0.00059
Inflammatory bowel disease	63	-0.5732	-1.8773	4.3E-05	0.00111	0.00092
Glutamatergic synapse	111	-0.5066	-1.8239	4.4E-05	0.00111	0.00092
Allograft rejection	35	-0.6609	-1.9448	0.00011	0.00254	0.0021
Taurine and hypotaurine metabolism	16	-0.7963	-1.9652	0.00011	0.00254	0.0021
Amphetamine addiction	69	-0.5521	-1.8401	0.00013	0.00279	0.0023
MicroRNAs in cancer	160	0.40191	1.6424	0.0002	0.004	0.0033
Autoimmune thyroid disease	50	-0.5818	-1.8419	0.00025	0.00461	0.0038
Staphylococcus aureus infection	87	-0.5019	-1.743	0.0003	0.00535	0.00441
Cocaine addiction	49	-0.5858	-1.8508	0.00034	0.00564	0.00465
Asthma	28	-0.677	-1.8853	0.00041	0.00644	0.00532
Signaling pathways regulating pluripotency of stem cells	140	0.39619	1.60953	0.00047	0.00706	0.00582
Cell adhesion molecules	147	-0.4349	-1.6294	0.0006	0.00868	0.00716
Malaria	47	-0.5783	-1.8155	0.00066	0.00895	0.00739
Nucleocytoplasmic transport	101	0.42755	1.66382	0.00067	0.00895	0.00739
Type I diabetes mellitus	41	-0.584	-1.7902	0.00073	0.00939	0.00775
Tuberculosis	177	-0.4053	-1.5455	0.00083	0.01024	0.00845
Graft-versus-host disease	38	-0.6079	-1.8309	0.00113	0.01345	0.0111
Th1 and Th2 cell differentiation	90	-0.4707	-1.6373	0.00121	0.01385	0.01143
Basal cell carcinoma	63	0.4654	1.64309	0.00143	0.01588	0.0131
T cell receptor signaling pathway	101	-0.4443	-1.5744	0.00239	0.02571	0.02121
Insulin resistance	105	-0.4493	-1.5999	0.00308	0.03207	0.02646
Viral myocarditis	57	-0.5092	-1.6451	0.00345	0.03485	0.02876
Protein digestion and absorption	100	0.39887	1.54667	0.00382	0.03706	0.03058
Tight junction	163	-0.3965	-1.5008	0.00398	0.03706	0.03058
Small cell lung cancer	92	0.40964	1.56086	0.00401	0.03706	0.03058
Rheumatoid arthritis	88	-0.4545	-1.5792	0.00414	0.03723	0.03072
Lipid and atherosclerosis	213	-0.37	-1.4395	0.005	0.04378	0.03612
NF-kappa B signaling pathway	99	-0.4281	-1.5101	0.00538	0.04497	0.0371
Long-term potentiation	67	-0.4755	-1.5762	0.0054	0.04497	0.0371
Bladder cancer	40	0.51751	1.68382	0.00555	0.04504	0.03716
NOD-like receptor signaling pathway	175	-0.3746	-1.431	0.00592	0.04683	0.03864
DNA replication	36	0.53172	1.68595	0.00605	0.04683	0.03864
